# Gut Microbiome Alterations Following Oral Serum-Derived Bovine Immunoglobulin Administration in the Management of Dysbiosis

**DOI:** 10.7759/cureus.75884

**Published:** 2024-12-17

**Authors:** Sabine Hazan, Guanhui Bao, Adriana Vidal, Adonis Sfera

**Affiliations:** 1 Gastroenterology, ProgenaBiome, Ventura, USA; 2 Research and Development, ProgenaBiome, Ventura, USA; 3 Research, ProgenaBiome, Ventura, USA; 4 Psychiatry, Patton State Hospital, San Bernardino, USA

**Keywords:** dietetics, dysbiosis, inflammatory bowel disease, irritable bowel syndrome, nutrition

## Abstract

Introduction: Inflammatory bowel disease (IBD) and irritable bowel syndrome (IBS) are chronic disorders of the gastrointestinal tract associated with gut microbiota dysbiosis and inflammation. Serum-derived bovine immunoglobulin (SBI) is used to manage IBS and IBD and has shown prebiotic-like effects in ex vivo models. Re-establishing a healthy gut microbiome with novel treatments like SBI could help treat the underlying causes of these diseases leading to higher and sustained patient response. The objective of this study was to assess whether supplementation with SBI would improve dysbiosis in IBD and IBS patients.

Methods: This cross-sectional, single-site study had each participant serving as their own control. Stool samples from 18 patients with either IBS or IBD were analyzed before and after SBI administration. The relative abundance of bacterial diversity was assessed using metagenomic next-generation sequencing-based profiling.

Results: Species diversity statistically significantly increased for measures of richness (Shannon index) (p < 0.0082) and evenness (Gini-Simpson index) (p < 0.0017). Phylum-level changes showed a 2.7-fold increase in *Actinobacteria* (p = 0.0181), 0.66-fold decrease in *Bacteroidetes* (p = 0.0401), and 0.38-fold decrease in *Proteobacteria* (p = 0.0071) after treatment with SBI. At the genus level, the relative abundances showed decreased *Alistipes* (p = 0.0121) and decreased *Bacteroides* (p = 0.0108) as well as increased *Bifidobacterium* (p = 0.0204), compared to pre-treatment levels. At the genus level, a 1.8-fold increase of *Bifidobacterium* *breve* (p = 0.0225) occurred upon treatment with SBI.

Conclusion: These findings confirm the prebiotic effects of SBI and suggest an additional mechanism of action in managing IBD and IBS symptoms. SBI re-establishes homeostasis in patients with IBD and IBS by decreasing *Proteobacteria* and increasing *Bifidobacteria *and species diversity. These insights highlight the promise of new therapeutic strategies for managing IBS and IBD by targeting dysbiosis and underscore the potential of personalized treatments based on a patient's gut microbiome profile.

## Introduction

Inflammatory bowel disease (IBD) and irritable bowel syndrome (IBS) are chronic, recurring gastrointestinal disorders that significantly impact patients' quality of life [1-3]. Both conditions cause symptoms such as abdominal pain, severe diarrhea, bloating, and weight loss [4,5]. There are no definitive cures for IBD/IBS, so current treatments focus on symptom relief. Many patients do not respond or lose responsiveness to medications such as corticosteroids, immunomodulators, biologics, and antibiotics. In IBD patients, approximately 40% have a primary non-response to tumor necrosis factor (TNF) inhibitors, and 23-46% lose response after one year [6]. In IBD patients, the partial response rate for the immunomodulator tacrolimus is 37% [7]. Corticosteroids have no proven efficacy in maintaining remission [7] and in IBS patients, rifaximin has only modest benefits over placebo [8,9]. New interventions are needed to target the underlying causes, offer broader efficacy across the patient population, and provide sustained effectiveness.

Patients with Crohn's disease (CD) and ulcerative colitis (UC) suffer from chronic inflammation of the gastrointestinal tract, associated with mucosal barrier dysfunction, dysregulated immune response, and dysbiosis. While IBS is multifactorial, involving gut-brain interactions, clinical characteristics include motility disturbances and visceral hypersensitivity without visible signs of damage or disease in the digestive tract. Both IBD and IBS are linked with dysbiosis of the gut microbiota in 70% and 73% of patients, respectively [10]. Several studies have tried to characterize the profile of gut bacteria in IBD patients, including those with CD and UC [10]; however, no clear consensus has been reached on specific changes in the gut microbiome that are associated with IBD. It has been reported that patients with active IBD tend to have lower microbial diversity, with more *Proteobacteria* and fewer *Firmicutes* [11,12]. Although, it remains unclear whether dysbiosis is the cause of IBD or a consequence of IBD [10,13,14]. Similarly, IBS is associated with patients having lower microbial diversity with more *Proteobacteria*, *Lactobacillus*, and *Bacteroides* and less *Clostridiales, Faecalibacterium*, and *Bifidobacterium* compared with controls [15]. Furthermore, the presence of *Clostridiales, Prevotella*, and methanogenic species has been identified as an IBS-specific microbiome signature correlating with severe symptoms [16,17]. The cause of this signature has not been explained by patients’ medications, diet, or genetics [16,17].

Recent scientific interest has emerged in the interplay between IBD/IBS treatments and the gut microbiome, given that in both conditions gut dysbiosis is observed. Dysbiosis is defined as the disruption to the microbiome resulting in an imbalance in the microbiota, changes in their functional composition and metabolic activities, or a shift in their local distribution [18]. A recent treatment used for IBD/IBS/dysbiosis is serum-derived bovine immunoglobulin/protein isolate (SBI), an oral medical food product enriched in immunoglobulins (>50% IgG, 5% IgM, and 1% IgA). It has been used successfully to safely reduce symptoms in people with diarrhea-predominant IBS [19] and in people with IBD [20].

SBI’s primary mechanism of action has been discussed extensively by Petschow et al. [21,22]. Its primary mode of action is the binding of IgGs to conserved microbial antigens such as lipopolysaccharide (LPS) and subsequent neutralization and steric exclusion from the lamina propria [21]. However, recent work by Van den Abbeele et al. has highlighted a secondary mechanism by which SBI is able to reduce inflammation, improve barrier integrity, and promote gut homeostasis - modulation of the gut microbiome [23-25]. When dosed at an equivalent of 5 g per day, SBI increased the abundance of species such as *Coprococcus comes* and *Dorea formicigenerans*, both of which are *Firmicutes*, which have reduced abundance in IBD. Additionally, SBI treatment of the microbiome led to significant increases in short-chain fatty acids, improved barrier function, and lowered inflammation upon LPS stress as measured by the pro-inflammatory molecules tumor necrosis factor-alpha (TNF-α) and CXCL10. Interestingly, the authors showed significant increases in tryptophan metabolites indole-3-carboxaldehyde, indole-3-propionic acid, and indole-3-acetic acid, which activate the aryl hydrocarbon receptor, which reduces pro-inflammatory cytokines such as TNF-α and increases gut barrier integrity through tight junctions [26,27]. Thus, the modulation of the microbiome could help explain how SBI is able to improve barrier function and reduce systemic inflammation [28] in patients with HIV-associated enteropathy. Additionally, serotonin deficiency is a prominent factor in IBS [29]. When SBI was used in an ex vivo model [23,24], it showed an increase in tryptamine, which induces the release of serotonin 5-HT, which stimulates gut motility by acting on enteric nervous system neurons that modulate gut motility [30]. While theex vivo results are quite promising, no previous study has focused on SBI's effects on the gut microbiome of IBS/IBD patients.

In this study, we used next-generation sequencing (NGS) to examine the composition of the gut microbiota in the stools of 18 IBD/IBS patients before and after 30 days of SBI treatment to test the hypothesis that specific microbial communities respond to this novel nutraceutical by re-establishing a healthy gut microbiome.

## Materials and methods

Study population and design

Any patient with a medical history and compatible presentation of IBD, IBS Rome IV, or gut dysbiosis was screened and allowed to participate in this study. This cross-sectional, single-site study had each participant serving as their own control. Stool samples were collected at baseline after which participants were prescribed to take 5 g SBI each day by mouth for 30 days. An additional stool sample was collected after treatment on day 30. Patients were excluded if they had (1) a history of bariatric surgery, total colectomy with ileorectal anastomosis, or proctocolectomy; (2) postoperative soma, ostomy, or ileoanal pouch; (3) participated in any experimental drug protocol within the prior 12 weeks; (4) treatment with total parenteral nutrition; and (5) change in concomitant medications during the trial. The Salus Institutional Review Board reviewed and approved the protocol (PR#002) and the study was registered at Clinicaltrials.gov (Identifier: NCT04031469). All participants provided written informed consent. Patients or the public were not involved in the design, conduct, reporting, or dissemination plans of our research.

Sample collection and processing

Stool samples were processed as previously described [31]. Briefly, participants were trained to collect fresh stool samples in a DNA/RNA Shield Fecal Collection Tube (Zymo Research, Tustin, CA), mixed thoroughly, and stored at -20°C until further processing. DNA was extracted and purified from each individual sample using the Qiagen DNA extraction kit (Hilden, Germany). The isolated DNA was quantitated utilizing a Quantus Fluorometer with the QuantiFluor ONE dsDNA kit (Promega Corporation, Madison, WI) and the DNA mass was normalized to 100 ng for library preparation. Prepared DNA samples underwent a shotgun metagenomic processing procedure of tagmentation, amplification, indexing, and purification. Upon completion of sequencing (Illumina NextSeq with 500/550 High-Output Kits V.2.5, Illumina, San Diego, CA), the raw data were streamed in real-time to Illumina’s BaseSpace cloud for FASTQ conversion.

Data analysis

The relative bacterial abundance at various taxonomic levels and Shannon and Gini-Simpson alpha diversity indices were determined using One Codex’s bioinformatics analysis pipeline and database (One Codex, San Francisco, CA). Given the typically non-normally distributed nature of microbiome data, statistical analysis employed the Wilcoxon signed-rank test. Significance was evaluated at a threshold of p < 0.05, and computations were conducted utilizing the R dplyr and Python seaborn libraries. To address multiple comparisons, the false positive rate of 5% was applied. Graphical representation of relative abundance, alpha diversity, and fold change of relative abundance values utilized median values, with error bars indicating the interquartile range (IQR) between the 1st and 3rd quartiles.

## Results

Demographics of the study population

Between October 2020 and April 2023, 18 participants were recruited at Ventura Clinical Trials (Ventura, CA). All 18 participants completed the study, and their demographics are shown in Table 1. The participants had a mean age of 41 (range: 3-80) years. Of the participants, 61% (n = 11) were women and 94% (n = 17) were of White race. Of the participants, 39% (n = 7) had Crohn's disease, 5.5% (n = 1) had ulcerative colitis, 50% (n = 9) had IBS, and 5.5% (n = 1) had dysbiosis. Nine patients (50%) were taking medication for their IBS/IBD conditions, which included mesalamine, Apriso, and budesonide.

**Table 1:** Patients characteristics. IBD: inflammatory bowel disease; IBS: irritable bowel syndrome.

Total participants (n = 18)	
Sex, n (%)	
Male	7 (38.9%)
Female	11 (61.1%)
Race, n (%)	
White	17 (94.4%)
Filipino	1 (5.6%)
Age, years (mean, range)	41 (3-80)
Condition, n (%)	
IBD Crohn’s disease	7 (38.9%)
IBD ulcerative colitis	1 (5.5%)
IBS	9 (50.0%)
Dysbiosis	1 (5.5%)
Concomitant medications	
For IBD/IBS	9 (50.%)
Other	4 (22.2%)
None	5 (27.8%)

SBI treatment modulates microbes at the phylum, genus, and species levels

**Figure 1:**
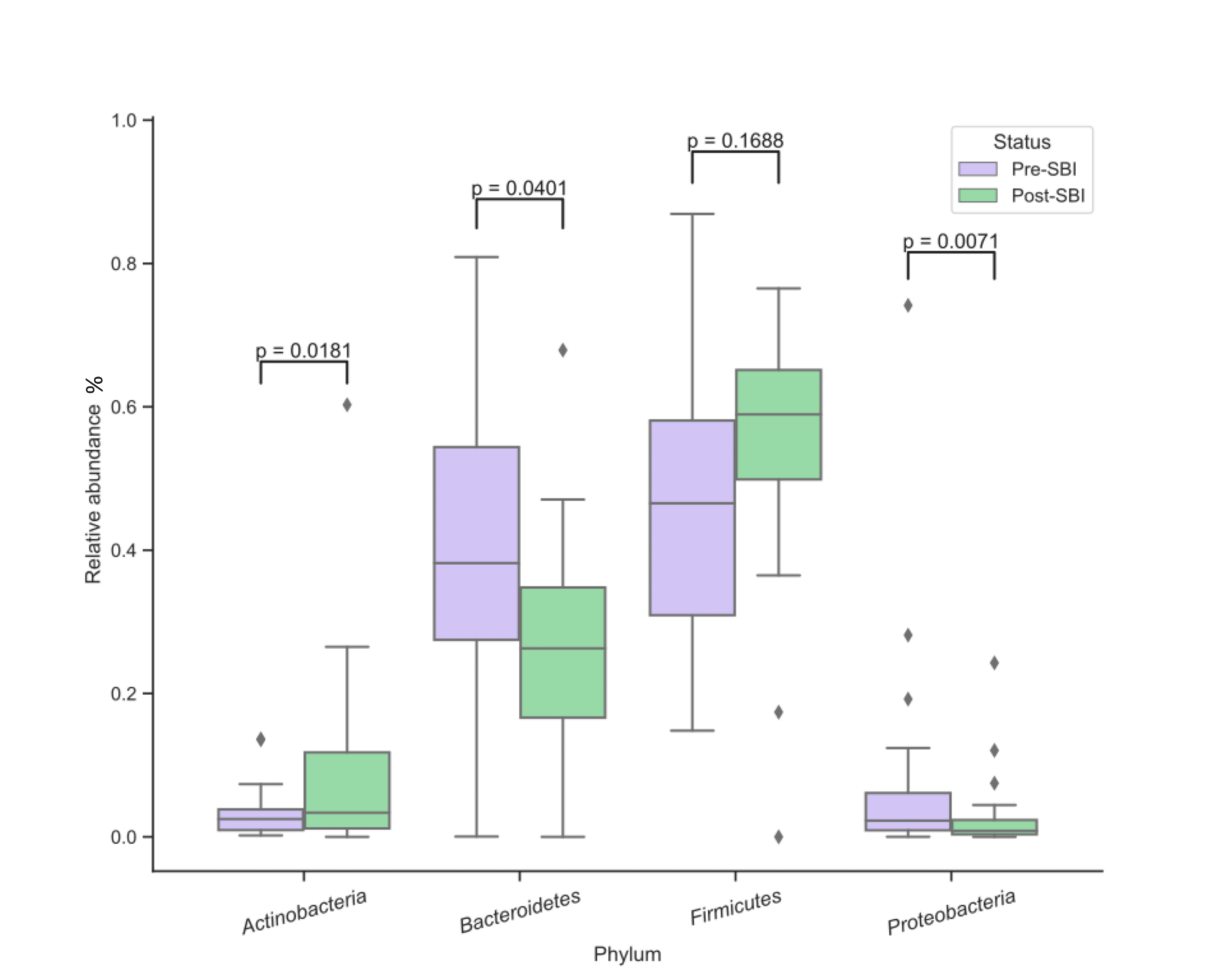
Relative abundances (%) of bacteria before and after SBI treatment at the phylum level. SBI: serum-derived bovine immunoglobulin.

**Figure 2:**
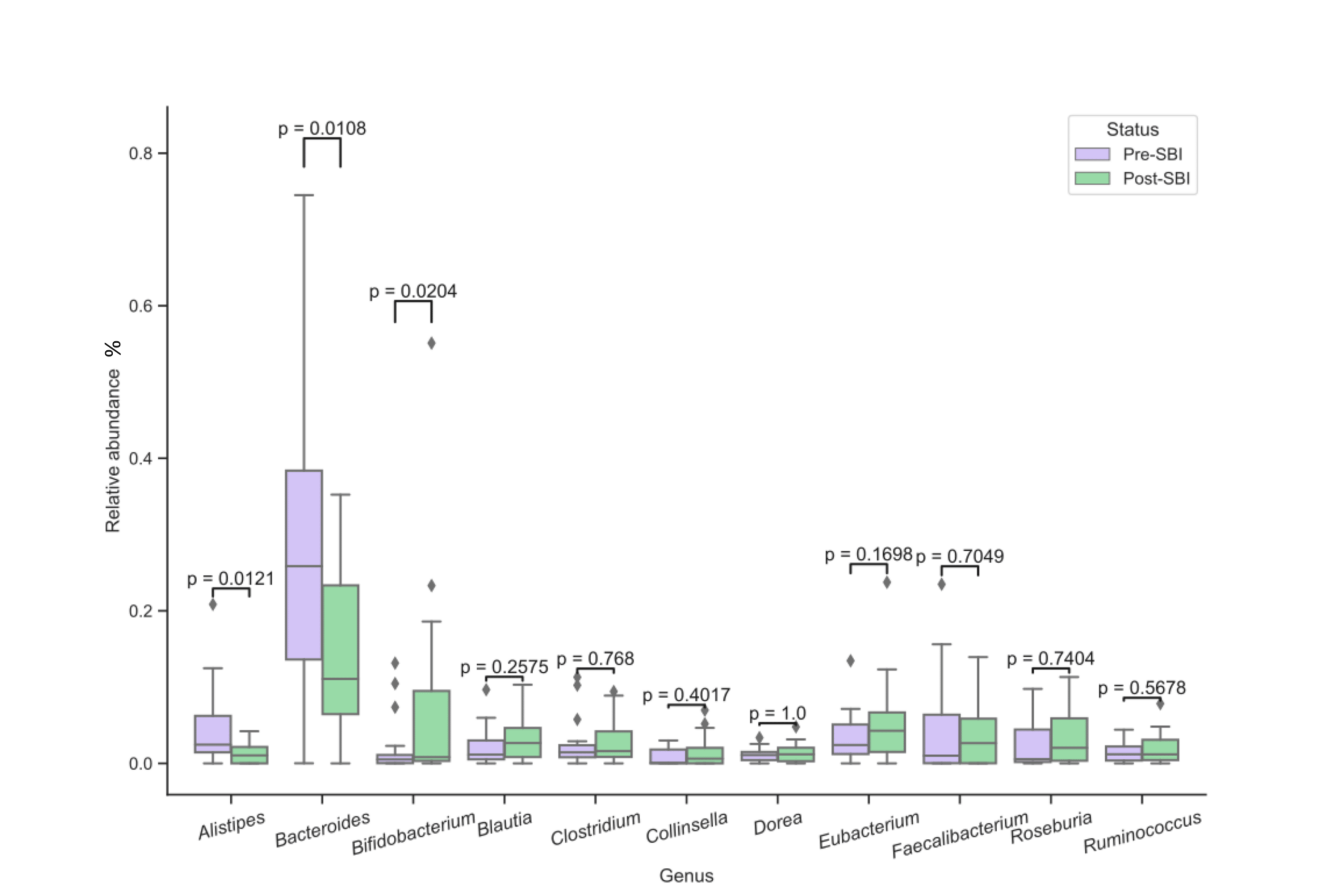
Relative abundances (%) of bacteria before and after SBI treatment at the genus level. SBI: serum-derived bovine immunoglobulin.

**Figure 3:**
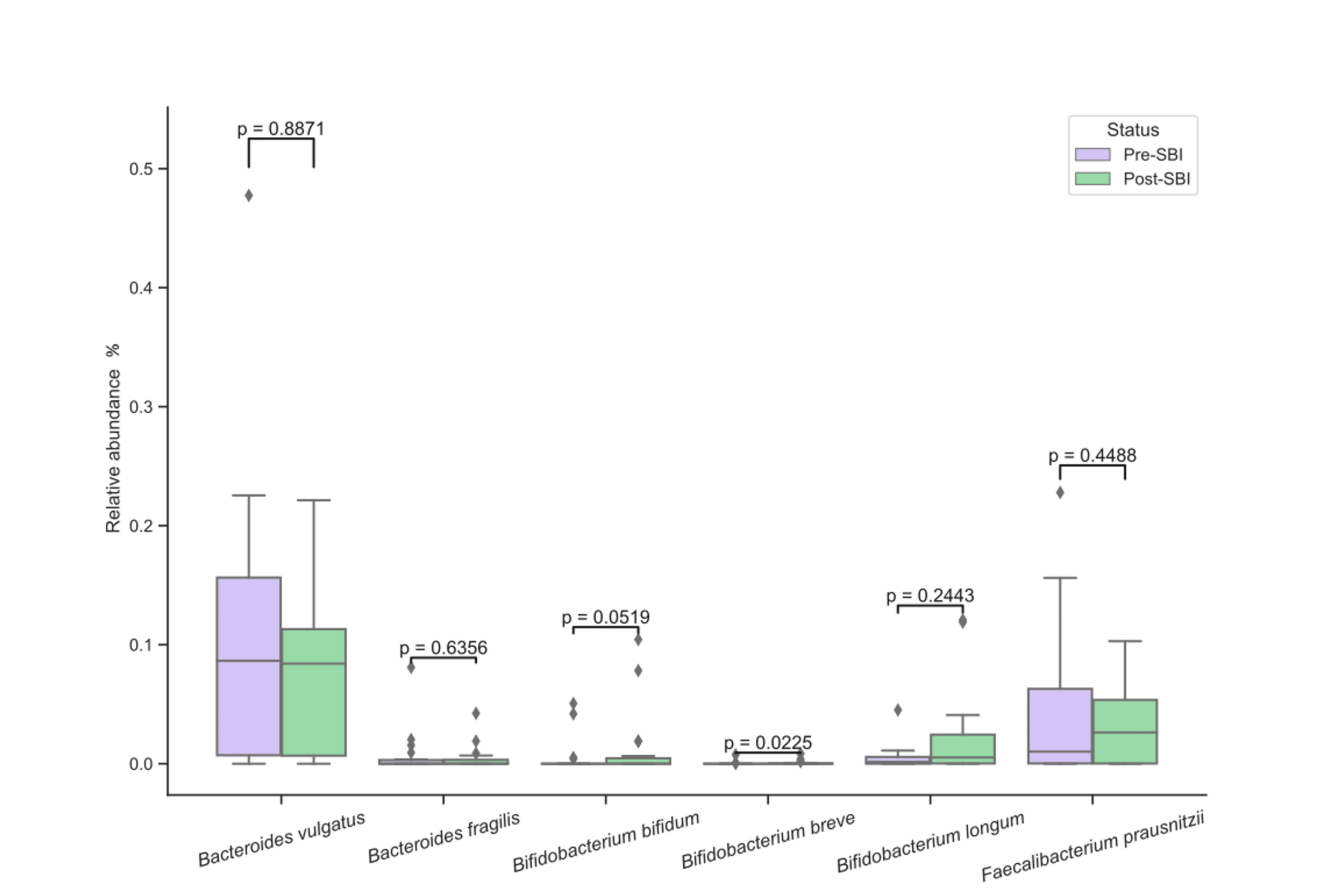
Relative abundances (%) of bacteria before and after SBI treatment at the species level. SBI: serum-derived bovine immunoglobulin.

To determine if 30 days of treatment with SBI modified the gut microbiota, the relative abundance of bacterial phyla, genera, and species were analyzed for each patient before and after treatment (Table 2). At the phylum level, the relative abundance of *Actinobacteria* increased 2.7-fold (p = 0.0181) after SBI treatment while *Bacteroidetes* decreased 1.5-fold (p = 0.0401), *Proteobacteria* decreased 2.6-fold (p = 0.0071), and *Firmicutes* showed no significant change (p = 0.1688) (Figure 1 and Table 2). Three genera had significant changes in relative abundance after SBI treatment: *Alistipes* decreased 3.4-fold (p = 0.0121), *Bacteroides* decreased 1.9-fold (p = 0.0108), and *Bifidobacterium* increased 3.6-fold (p = 0.0204) (Figure 2 and Table 2). For species, only *Bifidobacterium breve* showed significant changes, with an increase of 1.8-fold (p = 0.0225) after SBI treatment (Figure 3 and Table 2). Overall, these results suggest that SBI modulates the microbiome and does so directionally across patients for some microbes.

**Table 2:** Relative abundance of gut microbes in pre- and post-SBI-treated patients. * Wilcoxon rank test; statistically significant p-values (<0.05). SBI: serum-derived bovine immunoglobulin.

Taxonomic level	Bacteria	Pre-SBI				Post-SBI				Fold change	P-value*
		Mean	SD	Median	IQR	Mean	SD	Median	IQR		
Phylum	Actinobacteria	0.0355	0.0399	0.0248	0.0289	0.0954	0.147	0.0337	0.106	2.69	0.0181
	Bacteroidetes	0.395	0.219	0.382	0.269	0.26	0.171	0.263	0.182	0.66	0.0401
	Firmicutes	0.47	0.223	0.465	0.272	0.547	0.192	0.589	0.153	1.16	0.1688
	Proteobacteria	0.0863	0.175	0.0226	0.052	0.0327	0.0592	0.0085	0.02	0.38	0.0071
Genus	Alistipes	0.047	0.0544	0.0246	0.0478	0.0137	0.014	0.0106	0.0215	0.29	0.0121
	Bacteroides	0.274	0.191	0.258	0.248	0.141	0.105	0.111	0.169	0.51	0.0108
	Bifidobacterium	0.0202	0.0386	0.00517	0.0108	0.0726	0.135	0.0082	0.0917	3.59	0.0204
	Blautia	0.0211	0.0253	0.0115	0.0249	0.0312	0.0268	0.0267	0.0382	1.48	0.2575
	Clostridium	0.0256	0.0318	0.0145	0.0158	0.0278	0.0298	0.0161	0.0335	1.09	0.768
	Collinsella	0.0096	0.0119	0.0007	0.0181	0.0152	0.0208	0.0061	0.0204	1.58	0.4017
	Dorea	0.0113	0.0091	0.0109	0.0109	0.0133	0.0124	0.0119	0.0177	1.18	1
	Eubacterium	0.0345	0.0337	0.0241	0.0387	0.0521	0.0555	0.0428	0.0518	1.51	0.1698
	Faecalibacterium	0.0427	0.0632	0.0101	0.0636	0.0393	0.0438	0.0265	0.0584	0.92	0.7049
	Roseburia	0.0274	0.0346	0.0056	0.0425	0.0332	0.0367	0.0204	0.0556	1.21	0.7404
	Ruminococcus	0.0151	0.0143	0.012	0.0184	0.0193	0.0203	0.0119	0.0267	1.28	0.5678
Species	Bacteroides vulgatus	0.101	0.118	0.0864	0.149	0.0746	0.068	0.0841	0.106	0.74	0.8871
	Bacteroides fragilis	0.007	0.0188	0	0.0031	0.0045	0.0103	0	0.0033	0.64	0.6356
	Bifidobacterium bifidum	0.0054	0.0146	0	0	0.0121	0.0289	0	0.0046	2.24	0.0519
	Bifidobacterium breve	0.0005	0.0017	0	0	0.0009	0.0021	0	0.0004	1.80	0.0225
	Bifidobacterium longum	0.0053	0.0103	0.0015	0.0057	0.0206	0.0371	0.0052	0.0242	3.89	0.2443
	Faecalibacterium prausnitzii	0.0421	0.062	0.0101	0.0627	0.0323	0.0329	0.0263	0.0535	0.77	0.4488

SBI treatment increases bacterial diversity

**Figure 4:**
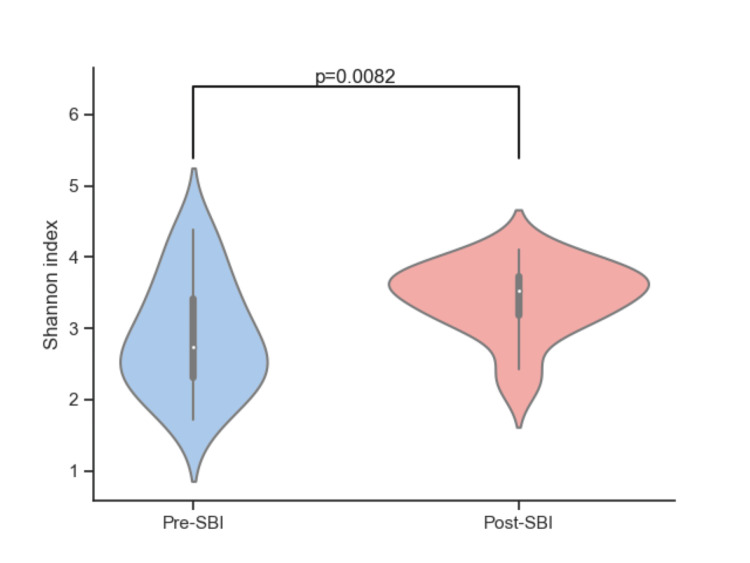
Alpha diversity at the genus level for pre-SBI and post-SBI using the Shannon diversity index. SBI: serum-derived bovine immunoglobulin.

Shannon and Gini-Simpson diversity indices were used to compare bacteria composition in pre- and post-SBI treatment. The Shannon diversity index considers both richness (the number of different species) and evenness (the relative abundance of different species) of the bacterial population but is weighted toward richness. Treatment with SBI significantly increased the Shannon diversity index of participants after 30 days of treatment with SBI (p = 0.0082, Figure 4). This improvement in the diversity index suggests an enhancement in both the variety and balance of microbial species in the gut following SBI treatment.

**Figure 5:**
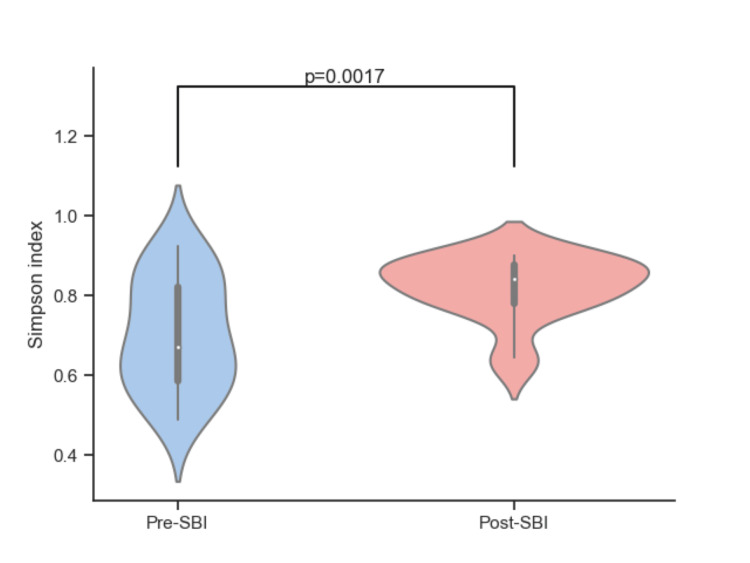
Alpha diversity at the genus level for pre-SBI and post-SBI using the Gini-Simpson diversity index. SBI: serum-derived bovine immunoglobulin.

The Gini-Simpson diversity index gives more weight to evenness and showed a significant increase post-SBI treatment (p = 0.0017, Figure 5). This improvement indicates a shift toward a more evenly distributed microbial community, with a decrease in the dominance of any one species. Together, these results suggest that supplementation with SBI positively modulates the gut microbiota.

## Discussion

One hundred years ago, the first causal relationship between IBD and dysbiosis was proposed when Bargen suggested diplostreptococci as a causative agent for ulcerative colitis [32]. Since then, additional microbes have been discovered to correlate with the disease state, but whether dysbiosis causes IBD/IBS or is solely a symptom remains disputed [13,14].Unfortunately, the Western diet and lifestyle have only increased the incidence of IBD/IBS, while decreasing microbial diversity [11,33]. While the incidence rates of IBD/IBS keep increasing, the currently available interventions do not treat the underlying conditions of dysbiosis, instead, they treat the symptoms with low and/or unsustainable response rates.

One treatment that may be affecting the gut microbiota is SBI. SBI is the active ingredient in a medical food that can be prescribed to help manage IBS [19] and IBD [20] and has been shown to have prebiotic-like effects [23,24]. In this study, we found that intervention with SBI for 30 days modulates the gut microbiome by altering the relative abundance of microbes at the phylum, genus, and species levels and increasing the microbial diversity. These observed microbiome modulations suggest that the positive clinical effects of SBI on managing IBS/IBD could be due to this additional mechanism of action, namely, correcting dysbiosis.

The significant changes observed in the gut microbiota's relative abundance and diversity following SBI administration provide compelling evidence of its potential role in modulating gut microbiota and managing IBS/IBD. Previous research indicates that the microbial taxa changes observed in this study play vital roles in maintaining gut health and modulating inflammation, which are crucial aspects of managing IBS/IBD [15-17]. At the phylum level, changes included an increase in *Actinobacteria*, and a decrease in *Bacteroidetes* and *Proteobacteria*, reflecting the re-establishment of a healthier gut microbiome. Specifically, inhibition of *Proteobacteria* could be beneficial as increases in that phylum are associated with active IBD [11,12].

At the genus level, *Alistipes* and *Bacteroides* decreased, and *Bifidobacterium* increased. The increases in abundance of *Bifidobacterium breve *at the species level further emphasize the impact of SBI on specific beneficial bacterial species. Considering* B. breve* has been shown to alleviate colitis in a mouse model of IBD [34], the observed increase of *B. breve* herein could explain the improvement of IBS/IBD symptoms observed in previous clinical studies [19,20]. When looking at these changes in the microbiome, the decreases in *Proteobacteria* and increases in *Bifidobacterium *by SBI could offset the increases and decreases associated with IBD of the respective phylum and genus.

The results of this study support and build upon the ex vivo work showing SBI has prebiotic-like effects. Both works showed changes to the microbiome modulated by SBI and specifically increases in *Firmicutes*, which rose to the level of significance in the ex vivo model but not in this cross-sectional study. The results of the ex vivo model did not show a significant increase in *Bifidobacterium*, yet in participants enrolled in this study, the increase in *Bifidobacterium* was significant. These differences are likely explained because healthy fecal donors were used in the ex vivo model versus the enrollment of participants with enteric dysfunction in this study. Interestingly, the ex vivo model showed that inulin exclusively stimulated *Bifidobacteriaceae* growth for donors classified as *Bacteroides*/*Firmicutes *enterotypes, which suggests a similar donor-specific effect could be observed for IBD and IBS patients taking SBI. These results also underscore the significance of personalized treatment approaches based on the patient's gut microbiome profile. These insights could pave the way for innovative therapeutic strategies in the management of IBS/IBD and other diseases associated with gut microbiota dysbiosis.

Lastly, it is worth noting the role that microbiome metabolites could play in the efficacy of SBI in IBD and IBS and how that relates to these results. The impact of tryptophan catabolites on improving gut homeostasis, reducing inflammation, and increasing barrier integrity via tight junctions, as mediated by the aryl hydrocarbon receptor was discussed in the introduction. Interestingly, *B. breve* has been shown to produce high levels of indole-3-lactic acid, which is reported to also maintain immune homeostasis [34,35]. This result could act as another layer to connect how SBI can reduce symptoms of IBS and IBD. Especially considering the reductions in TNF-α upon LPS challenge due to modulation of the ex vivo microbiome by SBI, which could be explained by the production of tryptophan catabolites. This result is made all the more intriguing because TNF-α inhibitors, such as infliximab and adalimumab, exert their effects by inhibiting TNF-α associated inflammation [1].

A limitation of this cross-sectional study is that these results provide a snapshot of SBI's impact on the gut microbiota and may not fully reflect the dynamic nature of the gut microbiome or the long-term effects of the treatment. In addition, analyses were not adjusted for confounders, such as diet and medications taken. Strengths of this study include the use of NGS to profile the gut microbes, which allowed us to obtain data at the genus and species level. Moreover, while the results of this study are promising, further research is required to elucidate the exact mechanisms through which SBI and bovine IgG influence the gut microbiota and to determine the clinical implications of these microbiome changes.

Overall, IBD/IBS has no known cure, but the prevalence of these diseases is increasing within modern culture. It is unclear if the dysbiosis associated with IBD/IBS causes the disease or is caused by the disease, but, in the clinical setting, correcting dysbiosis plays a key role in patient symptom management and return to health. SBI is known to help manage symptoms associated with IBD/IBS. This work highlights a novel mechanism of SBI to modulate the gut microbiome in addition to its primary mechanism of binding and exclusion. Together, these mechanisms showcase how SBI can reduce inflammation within the gut of IBD/IBS patients and suggest that SBI could be used to manage gut dysbiosis in other diseases. These insights into the modulation of specific phylum, genus, and species could pave the way for innovative therapeutic strategies in the management of IBS/IBD and other diseases associated with gut microbiota dysbiosis.

## Conclusions

This study shows that SBI may help re-establish gut homeostasis in patients with IBD and IBS. The findings herein described suggest that SBI supplementation may exert prebiotic effects by decreasing gut dysbiosis in patients with IBD and IBS via lowering gut *Proteobacteria* levels and increasing *Bifidobacteria*. Furthermore, SBI supplementation also helped improve gut bacteria species diversity in these patients. Larger prospective studies are needed to confirm these findings.
